# Supplementary figures and images for: Loss of Wnt16 Leads to Skeletal Deformities and Downregulation of Bone Developmental Pathway in Zebrafish
Source: Int J Mol Sci. 2021 Jun 22;22(13):6673. doi: 10.3390/ijms22136673 (PMC8268848; doi:10.3390/ijms22136673)

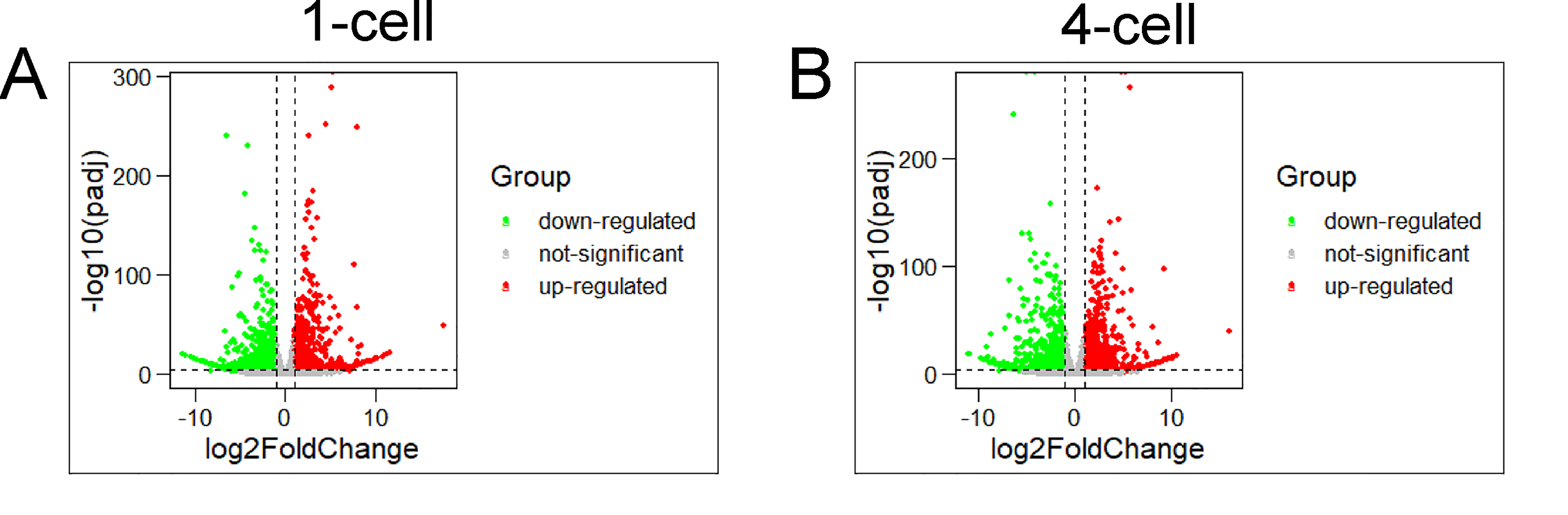

Supplement: Supplementary file 1 [file ijms-22-06673-s001.zip › Supplementary Materials/Figure S1. Volcano diagram and heat map of differentially expressed genes..png]

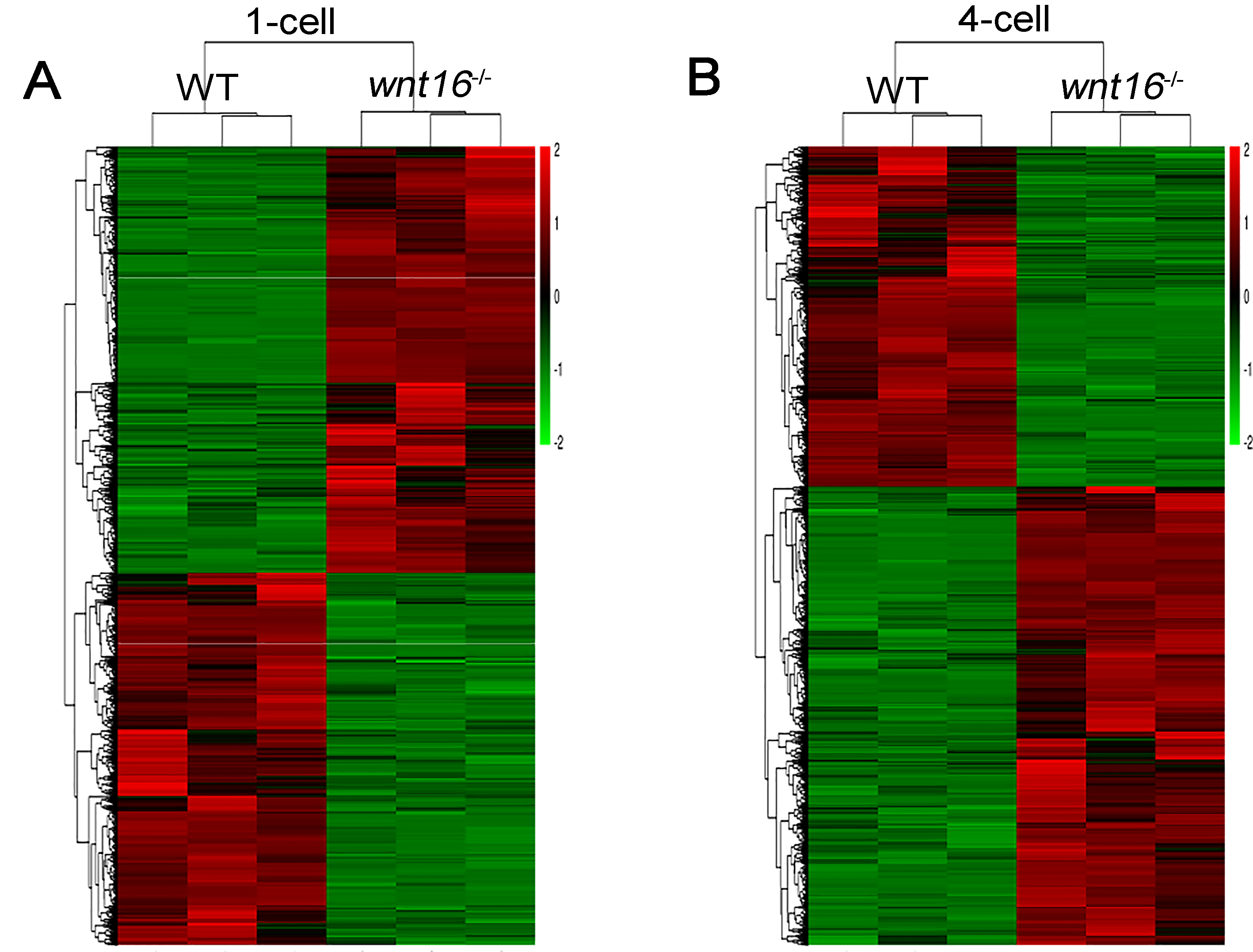

Supplement: Supplementary file 1 [file ijms-22-06673-s001.zip › Supplementary Materials/Figure S2. Clustering heat map of differentially expressed genes..png]

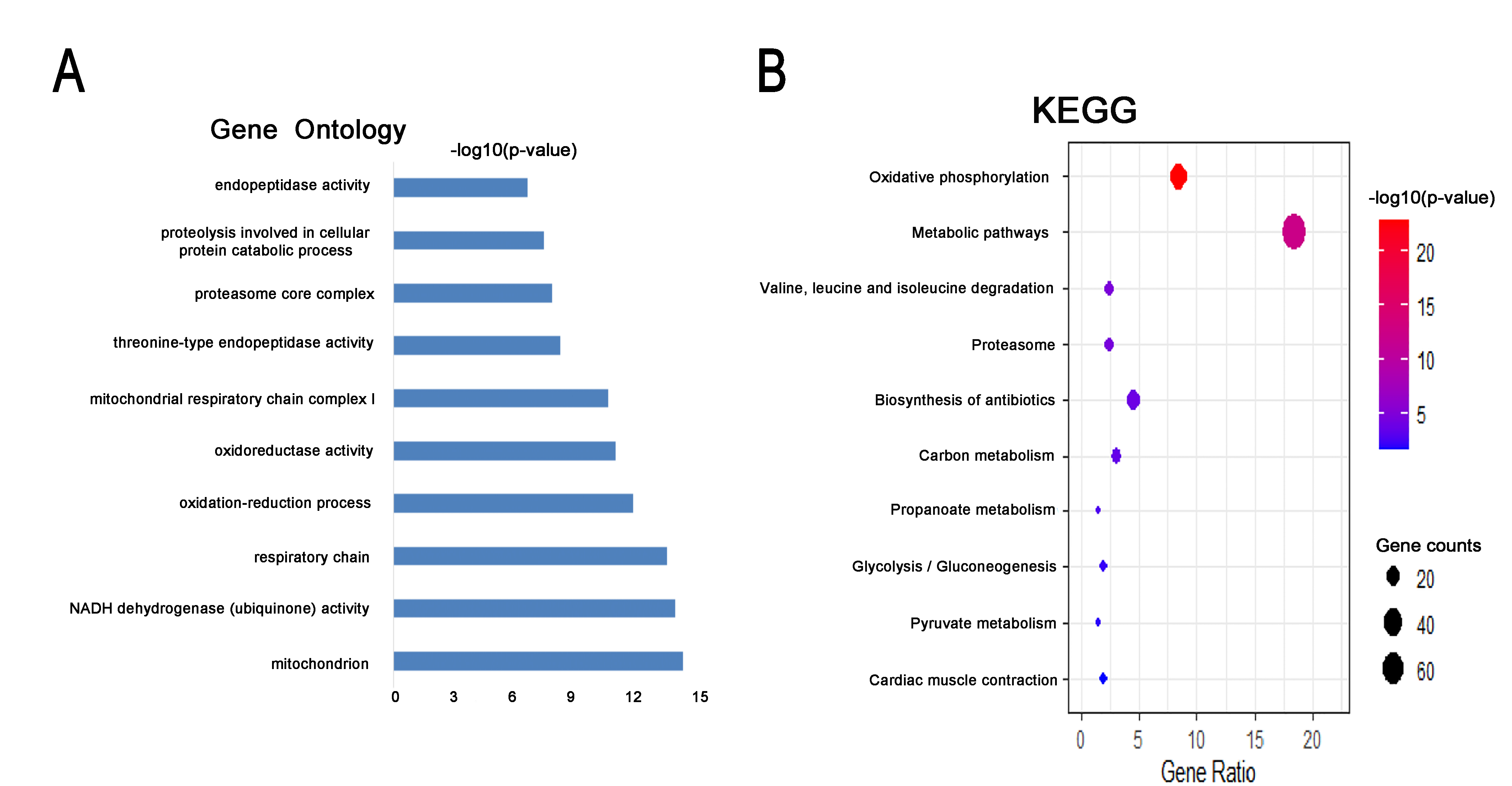

Supplement: Supplementary file 1 [file ijms-22-06673-s001.zip › Supplementary Materials/Figure S3. GO enrichment and KEGG signaling pathway analysis of co-expression up-regulated DEGs..png]
